# Post-treatment FDG PET-CT in head and neck carcinoma: comparative analysis of 4 qualitative interpretative criteria in a large patient cohort

**DOI:** 10.1038/s41598-020-60739-3

**Published:** 2020-03-05

**Authors:** Jim Zhong, Moses Sundersingh, Karen Dyker, Stuart Currie, Sriram Vaidyanathan, Robin Prestwich, Andrew Scarsbrook

**Affiliations:** 1 Department of Radiology, Leeds Teaching Hospitals NHS Trust, Leeds, UK; 2 Department of Nuclear Medicine, St James’s Institute of Oncology, Leeds, UK; 3 Department of Clinical Oncology, St James’s Institute of Oncology, Leeds, UK; 4 Leeds Institute of Medical Research at St James’s, Faculty of Medicine & Health, University of Leeds, Leeds, UK

**Keywords:** Cancer imaging, Head and neck cancer

## Abstract

There is no consensus regarding optimal interpretative criteria (IC) for Fluorine-18 fluorodeoxyglucose (FDG) Positron Emission Tomography – Computed Tomography (PET-CT) response assessment following (chemo)radiotherapy (CRT) for head and neck squamous cell carcinoma (HNSCC). The aim was to compare accuracy of IC (NI-RADS, Porceddu, Hopkins, Deauville) for predicting loco-regional control and progression free survival (PFS). All patients with histologically confirmed HNSCC treated at a specialist cancer centre with curative-intent non-surgical treatment who underwent baseline and response assessment FDG PET-CT between August 2008 and May 2017 were included. Metabolic response was assessed using 4 different IC harmonised into 4-point scales (complete response, indeterminate, partial response, progressive disease). IC performance metrics (sensitivity, specificity, positive predictive value (PPV), negative predictive value (NPV), accuracy) were compared. Kaplan-Meier and Cox proportional hazards regression analyses were performed for survival analysis. 562 patients were included (397 oropharynx, 53 hypopharynx, 48 larynx, 64 other/unknown primary). 420 patients (75%) received CRT and 142 (25%) had radiotherapy alone. Median follow-up was 26 months (range 3–148). 156 patients (28%) progressed during follow-up. All IC were accurate for prediction of primary tumour (mean NPV 85.0% (84.6–85.3), PPV 85.0% (82.5–92.3), accuracy 84.9% (84.2–86.0)) and nodal outcome (mean NPV 85.6% (84.1–86.6), PPV 94.7% (93.8–95.1), accuracy 86.8% (85.6–88.0)). Number of indeterminate scores for NI-RADS, Porceddu, Deauville and Hopkins were 91, 25, 20, 13 and 55, 70, 18 and 3 for primary tumour and nodes respectively. PPV was significantly reduced for indeterminate uptake across all IC (mean PPV primary tumour 36%, nodes 48%). Survival analyses showed significant differences in PFS between response categories classified by each of the four IC (p <0.001). All four IC have similar diagnostic performance characteristics although Porceddu and Deauville scores offered the best trade off of minimising indeterminate outcomes whilst maintaining a high NPV.

## Introduction

Head and neck squamous cell carcinoma (HNSCC) is the sixth leading cancer by incidence worldwide, accounts for >90% of head and neck cancer, has an annual incidence of over 550,000 with around 300,000 deaths each year^1^.

The 5-year overall survival for HNSCC is 40–50% and more than two thirds of patients present with locally advanced disease mandating accurate staging^2^. Recurrence rates as high as 60% within 2 years of treatment have been reported with 20–30% of patients developing distant metastatic disease^3^. For loco-regionally advanced HNSCC, chemoradiotherapy (CRT) has increasingly become a standard of care.

Fluorine-18 fluorodeoxyglucose (FDG) positron emission tomography – computed tomography (PET-CT) is central to characterising loco-regional and distant disease at initial staging and has an increasing role in post-treatment response assessment^4^. Randomised controlled trial data has shown that PET-CT performed post CRT is an accurate and cost-effective technique for assessing response and can spare 80% of patients from unnecessary neck dissection^5^. Post-treatment related changes in the neck can make assessment difficult in some cases, with evidence suggesting that human papilloma virus(HPV)-positive HNSCC behaves differently to HPV-negative disease, the specific test characteristics of PET-CT for assessing treatment response in HPV-negative HNSCC remains unclear^5,6^.

Semi-quantitative methods of treatment response assessment using standardised uptake value (SUV) have not been shown to be accurate at predicting patient outcome which has led to the development of more reproducible qualitative interpretative criteria (IC) to assess post-treatment response^7–11^. Heterogeneity in criteria used for assessment also limits comparison between different response assessment studies.

More recently, qualitative IC such as the Porceddu, Hopkins and Deauville scoring systems (Table 1) have been developed and validated in HNSCC response assessment^12–14^. These rely on visual inspection of the relative difference in tumour metabolism compared to surrounding normal tissue and/or background uptake, which in the case of Hopkins is the internal jugular vein and in Deauville, is the mediastinal blood pool. Both Hopkins and Deauville criteria use 5-point scales, however scores 1 and 2 in both categories effectively represent a complete metabolic response. Porceddu criteria employ a 3-point scale which classifies scans as positive, negative or equivocal based on whether there is FDG activity greater than adjacent normal tissues and/or liver^12^.

**Table 1:** Response interpretation criteria and explanation of each category.

Score	FDG Uptake	Category
Porceddu		
1	No residual FDG activity above background or diffuse uptake in the absence of a corresponding suspicious structural abnormality	Negative
2	FDG activity greater than adjacent normal tissues but below background liver activity	Equivocal
3	Focal uptake corresponding to a structural abnormality of greater intensity than background liver	Positive
**Hopkins**		
1	Minimal uptake (<internal jugular vein (IJV))	Complete metabolic response (CMR)
2	Minimal uptake (>IJV but <liver)	Probably CMR
3	Diffuse uptake (>IJV and liver)	Probably post radiation inflammation
4	Moderate focal uptake (>liver)	Probably persistent tumour
5	Intense focal uptake (>liver)	Persistent tumour
**Deauville**		
1	No uptake	CMR
2	Minimal uptake (<mediastinal blood pool (MBP))	Probably CMR
3	Low-grade uptake (>MBP but <liver)	Probably post radiation inflammation
4	Moderate focal uptake (>liver)	Probably persistent tumour
5	Intense focal uptake (>2 × liver) or new lesions	Persistent tumour
**NI-RADS**		
0	Incomplete imaging	Incomplete
1	No abnormal FDG uptake/Diffuse linear mucosal enhancement after radiation	No recurrence
2	Focal mucosal enhancement, but non mass-like or focal mild to moderate mucosal FDG uptake	Low suspicion
3	New or enlarging primary mass or lymph node or Intense focal uptake	High suspicion
4	Pathologically proven or definite radiological and clinic progression	Definite recurrence

Several studies have reported that qualitative assessment methods are useful for predicting regional control and can help minimise the number of equivocal scan results^13,15,16^. In 2016, the American College of Radiology convened a Neck Imaging Reporting and Data Systems (NI-RADS) Committee who have developed a template to help distinguish benign post-treatment changes and residual or recurrent tumour^17^.

Currently there is no clear consensus regarding the optimal IC to use in this clinical scenario. Classifying ‘equivocal’ cases varies depending on which IC is used and differences remain in how these patients are subsequently managed, for example, undergoing invasive neck dissection or further follow-up imaging and clinical examination given the difficulty in differentiating a benign post-treatment response or residual/recurrent tumour^18^.

The primary objective of this study was to assess comparative accuracy and prognostic ability of the 4 different IC (NI-RADS, Porceddu, Hopkins and Deauville) in a large cohort of HNSCC patients treated with curative-intent (chemo)radiotherapy for predicting local and regional disease control and progression free survival (PFS).

## Methods

### Patient cohort

The study involved retrospective analysis of a prospective database performed under a waiver of informed consent and ethics approval by the Institutional Review Board. Prospective consent was obtained from all patients for use of their PET-CT imaging data in research and service development projects. Consecutive patients with histologically confirmed HNSCC treated at a tertiary referral centre between August 2008 and May 2017 with curative-intent non-surgical treatment (radiotherapy alone or chemoradiotherapy) who had undergone baseline and response assessment FDG PET-CT. Our institutional protocol is for response assessment PET-CT to be performed approximately 4 months after treatment. Demographics, baseline characteristics, staging, treatment and outcome details were retrieved from the institutional electronic patient record (PPM+, Leeds, United Kingdom). Exclusion criteria included: patients with nasopharyngeal carcinoma; previous resection of primary or nodal disease; prior radiotherapy; FDG PET-CT only performed at baseline or for response assessment

### Treatment

Patients were treated with either three-dimensional (3D)-conformal radiotherapy or intensity-modulated radiotherapy (IMRT), which was gradually introduced into routine clinical practice from 2010. The 3D-conformal radiotherapy technique^19^ and IMRT^20^ have been previously described. Institutional protocols were followed with a radical treatment dose of 70 Gy in 35 fractions over 7 weeks or 65 Gy in 30 fractions over 6 weeks, with lower doses to prophylactic dose regions (54–63 Gy in 35 fractions over 7 weeks).

Induction chemotherapy with docetaxel, cisplatin and 5-fluorouracil (TPF) or cisplatin and 5-fluorouracil (PF) were delivered to a proportion of patients as previously described^21^. Concurrent chemotherapy routinely consisted of cisplatin 100 mg m^−2^ at days 1 and 29.

### Response assessment and follow-up

Tumour response was routinely assessed by clinical examination, naso-endoscopy where appropriate and FDG PET-CT approximately 4 months after completing treatment. Examination under anaesthetic and biopsies were performed at clinical discretion following response assessment. In general, patients who achieved a complete metabolic response did not undergo biopsy. Patients with less than a complete response were managed on an individual basis based upon discussion at a multidisciplinary team meeting. Subsequently, patients were followed up with physical examination and flexible endoscopy every 6–8 weeks in the first year after treatment, every 3 months for an additional 2 years and every 6 months until discharge at 5 years^22^.

### PET-CT technique

FDG PET-CT examinations prior to June 2010 were performed on a 16-slice Discovery STE PET-CT scanner (GE Healthcare, Chicago, Illinois, USA) and from June 2010 to October 2015 on a 64-slice Gemini TF64 scanner (Philips Healthcare, Best, Netherlands), After October 2015 all scans were performed on a 64-slice Discovery 710 scanner (GE Healthcare, Chicago, Illinois, USA). Serum blood glucose was routinely checked and if >10 mmol/L scanning was not performed. Patients fasted for 6 hours prior to intravenous Fluorine-18 FDG injection (dose varied according to patient body weight). PET acquisition from skull vertex to upper thighs was performed 60 minutes after tracer injection. A silence protocol was employed in the uptake period following tracer injection to minimize physiological tracer activity within the head and neck region. The CT component was performed according to a standardized protocol (without the use of iodinated contrast medium) with the following settings: 120 kV; auto-modulated mAs; tube rotation time, 0.5 seconds per rotation; pitch, 6; section thickness, 2.5 mm (to match the PET section thickness).

Patients maintained normal shallow respiration during the CT acquisition. Images were reconstructed using a standard ordered subset expectation maximization (OSEM) algorithm with CT for attenuation correction. Both non-attenuation-corrected and attenuation corrected datasets were reconstructed.

### Image analysis

**Figure 1:**
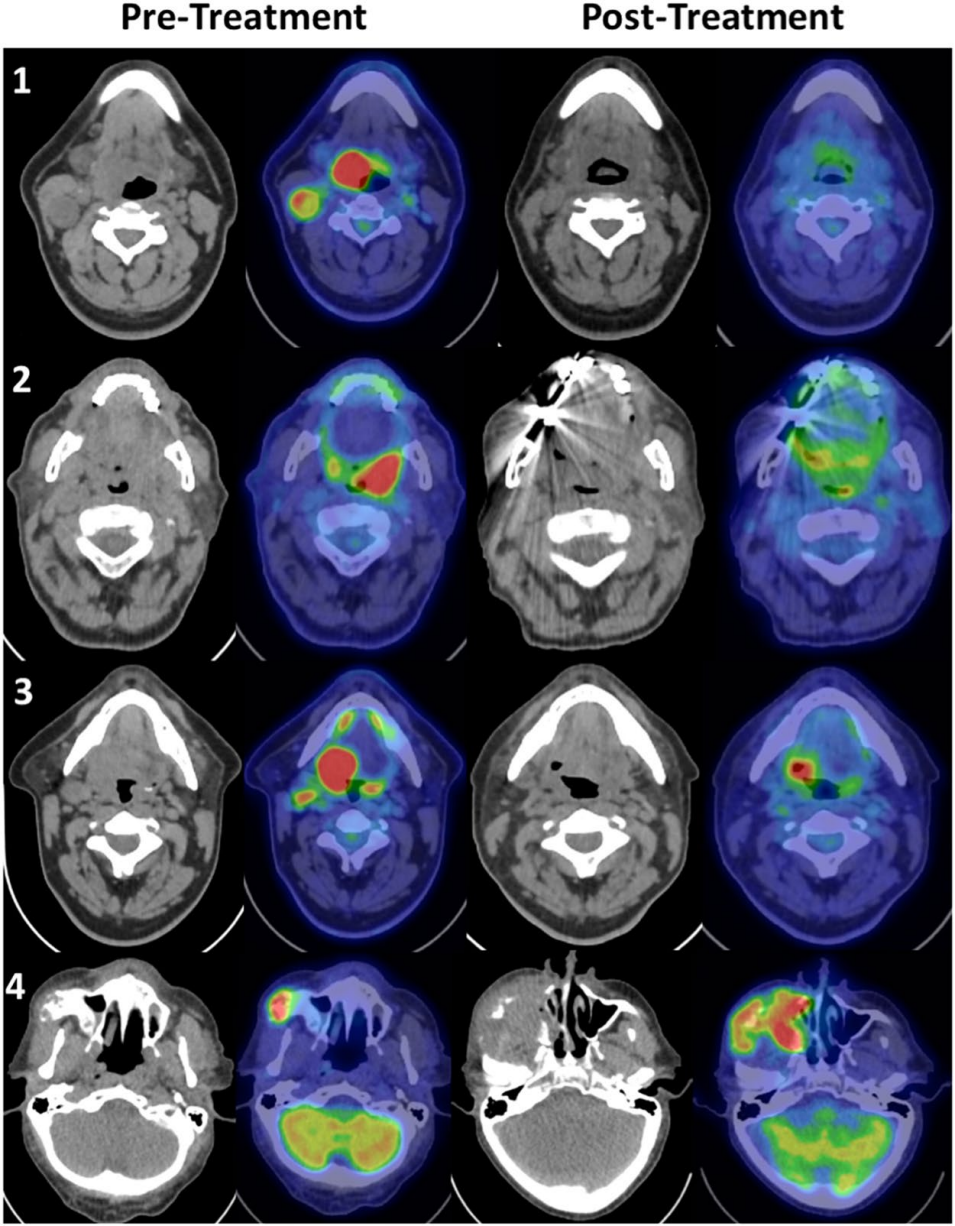
Representative cases illustrating post-harmonisation interpretative categories (1 to 4) pre and post-treatment. Row 1 – Complete response, Row 2 – Indeterminate, Row 3 – Partial response, Row 4 – Progressive disease.

All response assessment PET-CT studies were evaluated by a trainee radiologist under supervision of a dual-accredited Radiologist & Nuclear Medicine Physician with 15 years’ experience of reporting oncological PET-CT using specialised software (Advantage Windows Version 4.5, GE Healthcare, Chicago, Illinois, USA) and each of the four IC were applied. To accurately compare all four response assessment scales, each scale was re-classified into a 4-point scale as shown in Table 2 with complete response, partial response, indeterminate and progressive disease categories. Representative examples of these 4 categories are shown in Fig. 1.

**Table 2:** Harmonisation process of each interpretative criteria into standardized 4-point scales.

Response category	NI-RADS	Porceddu	Hopkins	Deauville
1. Complete response	1	0	1 + 2	1 + 2
2. Indeterminate	2	1	3	3
3. Partial response	3	2	4	4
4. Progressive disease	4	3	5	5

### Clinical follow-up

Follow-up was defined from final fraction of radiotherapy treatment. Disease status post-treatment was determined from pathology and/or radiology correlation with review of electronic patient records for clinical outcome. In patients who did not receive a biopsy/surgical intervention, serial negative physical examinations over the follow-up period and any relevant imaging investigations were used as confirmation of disease-free status.

### Statistical analysis

Survival and recurrence time was defined from final fraction of radiotherapy treatment. Diagnostic performance metrics for each IC: sensitivity, specificity, positive predictive value (PPV), negative predictive value (NPV) and overall accuracy applied to both primary tumour and nodes were calculated. Performance in sub-groups including HPV-positive oropharyngeal cancers (OPC), HPV-negative OPC and hypopharynx/larynx cancers were analysed.

Univariate association between recurrence (local and/or regional and/or distant) and each adjusted response assessment score (1–4) was estimated by the Chi-squared test. Kaplan-Meier analysis and Cox proportional hazards regression analyses were performed for each IC to assess cumulative progression free survival (PFS) and time to death (overall survival, OS) or progression. Log-rank testing was used to compare survival between the response categories within each IC. Receiver-operating characteristic (ROC) curve analysis was performed for each IC. The statistical significance level was set at P < 0.05. All statistical tests were performed using SPSS for Windows software (Version 21.0; IBM Corp., Armonk, New York, USA).

## Results

### Patient characteristics

A total of 562 patients were included in analysis. Detailed patient characteristics are provided in Table 3. Median age was 58 years (range 24–84). The median (range) of baseline tumour SUV_max_ and nodal SUV_max_ were 11.0 (0–53) and 8.1 (0–34) respectively. Median response tumour SUV_max_ was 1.7 (0–14.3) and nodal SUV_max_ was 1.0 (0–12.5).

**Table 3:** Patient characteristics.

Characteristics		Number	%
Gender	Male	423	75
	Female	139	25
Smoking	Smoker	205	36.5
	Ex-smoker	176	31
	Never smoked	149	26.5
	Not recorded	32	6
Tumour site	Paranasal sinus	9	2
	Oral cavity	7	1
	Oropharynx	397	71
	Hypopharynx	53	9
	Larynx	48	8.5
	Unknown	48	8.5
Grade	Well differentiated	7	1
	Moderately differentiated	118	21
	Poorly differentiated/ basaloid	364	65
	Undifferentiated	6	1
	Not recorded	67	12
HPV status	Positive	228	40
	Negative	55	10
	Not recorded	279	50
T stage	TX	47	8
	T1	101	18
	T2	188	34
	T3	120	21
	T4	106	19
N stage	N0	81	14
	N1	61	11
	N2a	42	8
	N2b	274	49
	N2c	99	18
	N3	5	1
Stage group (AJCC)	I	4	1
	II	24	4
	III	80	14
	IV	454	81
Treatment	CRT	420	75
	RT only	142	25

### Outcomes

Median follow-up period was 26 months (range 3–148 months). Median time from end of treatment to response assessment PET-CT was 17 weeks (range 6–31 weeks). 2-year survival outcomes were as follows: PFS 73%; OS 79%; local PFS 89%; regional PFS 85%; distant PFS 88%.

130 patients (23%) died in the study period with 432 patients (77%) alive at the time of analysis. 13 patients (2%) died within 6 months of treatment; one from a sudden cardiac event, two from tumor haemorrhage and 10 from disease progression. During follow-up, 156 patients (28%) developed progressive disease, 31 (20%) at the primary tumour site only (local failure), 42 (27%) at a regional nodal site only (regional failure), 16 (10%) at both the primary tumour and nodal site (loco-regional failure) without distant metastases and 35 (22%) had distant metastases only. 32 patients (21%) had local and/or regional failure with distant metastases. 11 cases (7%) of progressive disease were biopsy proven, 144 (92%) were based on radiology and 1 was a clinical diagnosis. 22 of 35 patients who developed distant metastases had these detected on response assessment PET-CT, 13 patients developed metastatic disease subsequently. Median time to loco-regional recurrence was 4 months (range 2–53).

**Figure 2:**
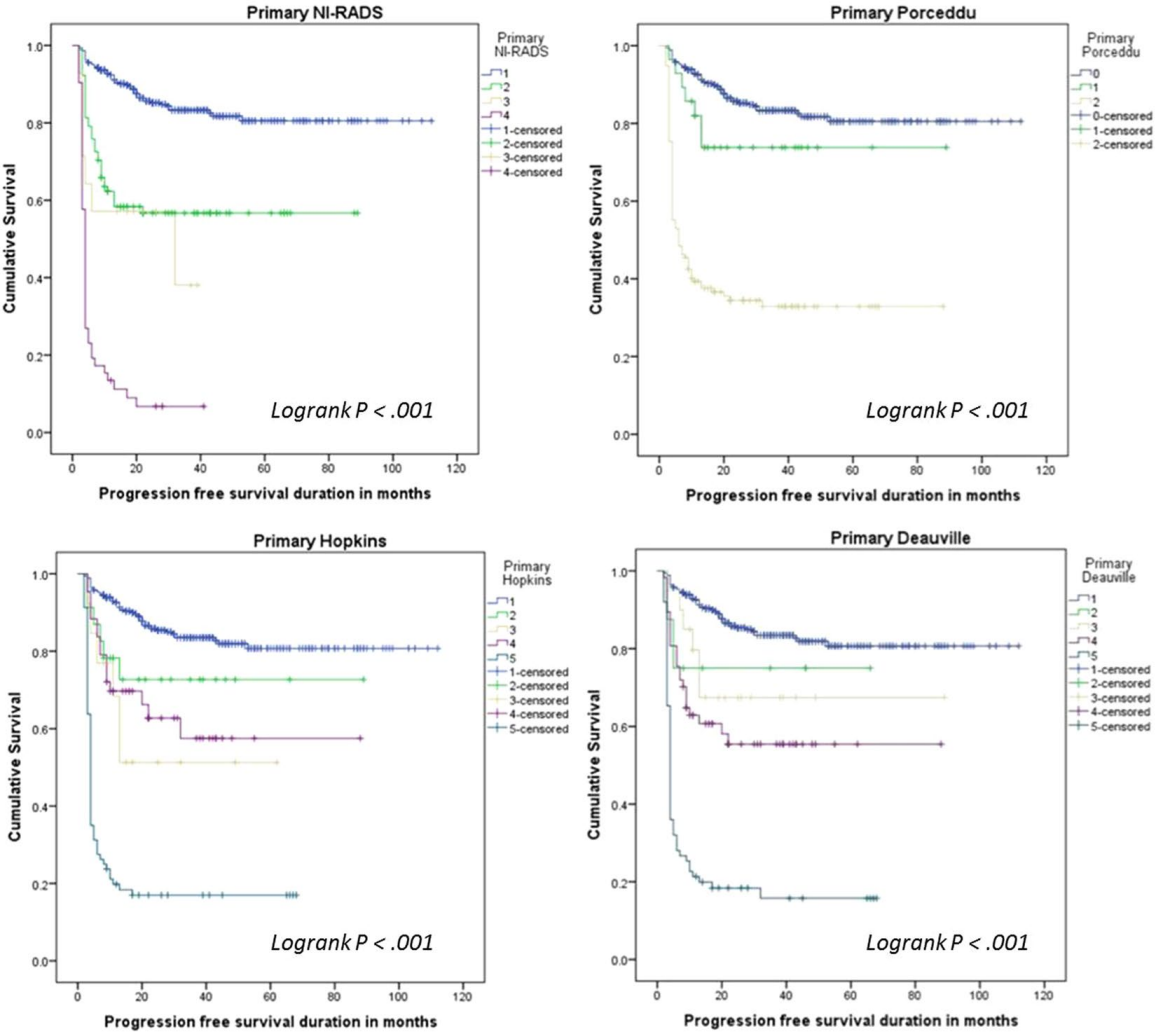
Kaplan-Meier plots of progression free survival based on each primary interpretative criteria before harmonisation. NI-RADS criteria (Categories 1–4), Porceddu criteria (Categories 1–3), Hopkins criteria (Categories 1–5) and Deauville criteria (Categories 1–5).

**Figure 3:**
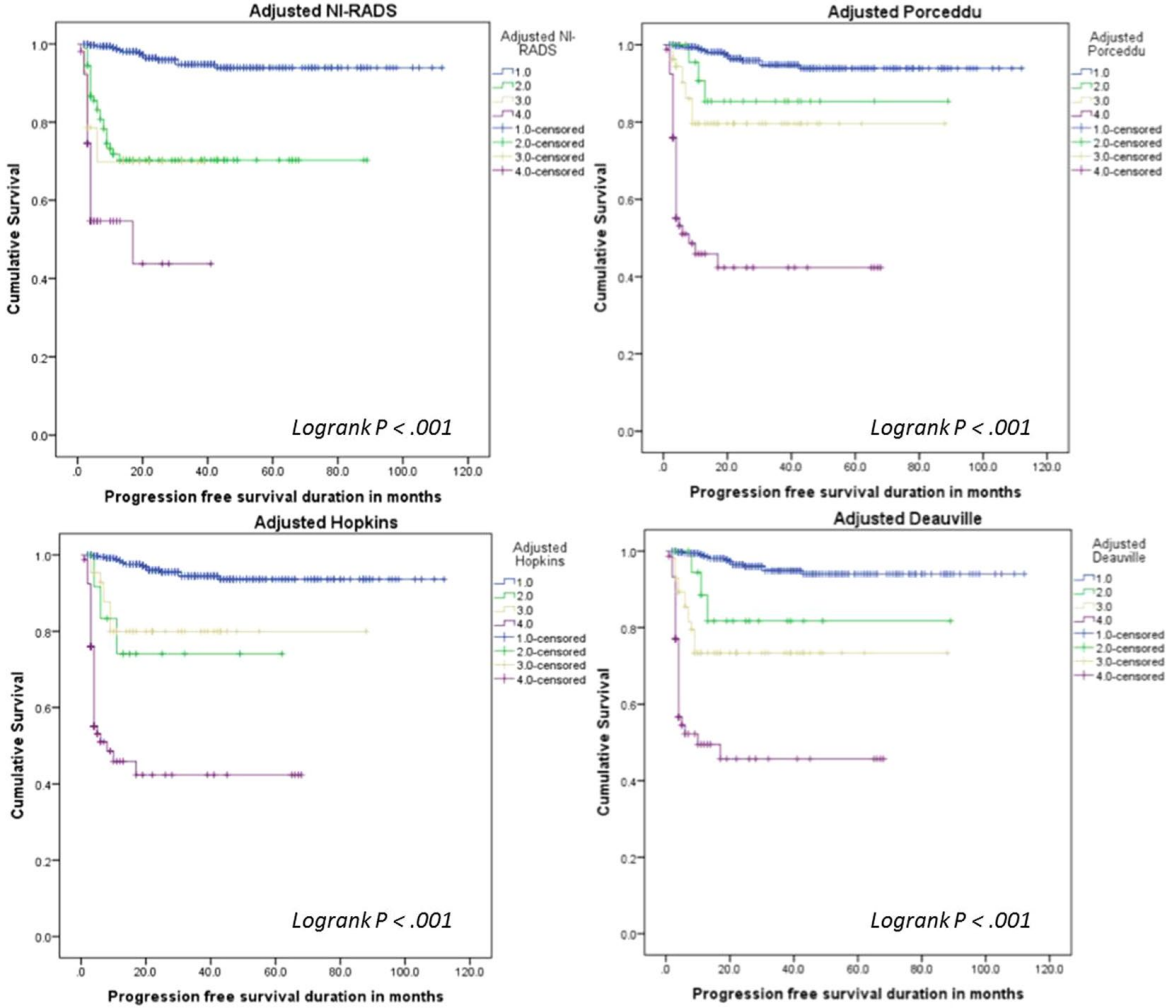
Kaplan-Meier plots of progression free survival based on each interpretative criteria post-harmonisation into 4-point scales.

Kaplan-Meier, the log-rank test and Cox proportional hazards regression analyses showed significant differences in PFS and OS between response categories classified by each of the four IC (p < 0.0001). Pairwise log-rank results provided in supplementary information. The survival curves pre and post harmonisation are shown in Figs. 2 and 3.

### Indeterminate cases

The number of indeterminate scores varied for each IC as shown in Table 4. With regards to primary tumour, NI-RADS classified 91 patients as indeterminate compared to 25 for Porceddu, 20 for Deauville and 13 for Hopkins. Overall, the NI-RADS IC scored more cases than the other 3 IC combined as indeterminate i.e. equivocal. Hopkins scored the fewest number of indeterminate cases.

**Table 4:** Indeterminate scores as categorised according to different interpretative criteria for each analysis group (All primary tumour, all node, HPV-positive OPC, HPV-negative OPC and hypopharynx/larynx sub-groups).

Group	NI-RADS		Porceddu		Hopkins		Deauville	
	N	R (%)	N	R (%)	N	R (%)	N	R (%)
All Tumour	91	38 (42%)	25	7 (28%)	13	6 (46%)	20	6 (30%)
All Node	55	32 (58%)	70	21 (30%)	3	3 (100%)	18	10 (56%)
HPV + ve OPC	32	11 (34%)	9	2 (22%)	4	2 (50%)	8	2 (25%)
HPV − ve OPC	9	5 (56%)	2	2 (100%)	1	1 (100%)	1	1 (100%)
Hypopharynx/Larynx	23	10 (43%)	2	1 (50%)	3	0 (0%)	2	1 (50%)

Key: N = total number of cases. R = number of cases with disease recurrence (percentage calculation of number of cases recurred as a proportion of total number of indeterminate cases for the group) OPC = oropharyngeal cancer.

### Diagnostic performance of interpretation criteria

The diagnostic performance of each IC in predicting disease control with regard to primary tumour, nodal disease, HPV-positive OPC, HPV-negative OPC and hypopharynx/larynx sub-groups are displayed in Table 5. The performance of each IC in predicting complete response and progressive disease in the indeterminate groups is shown in Table 6.

**Table 5:** Diagnostic performance of interpretative criteria for prediction of complete response and progressive disease applied to all primary tumours, all nodal disease, HPV-positive OPC, HPV-negative OPC and Hypopharynx/Larynx sub-groups. Mean values for each diagnostic performance metric across all 4 IC are also provided. () = number of cases provided.

	NI-RADS	Porceddu	Hopkins	Deauville	Mean
All Primary Tumour					
Sensitivity	47.06	55.46	52.8	52.99	52.1
Specificity	98.72	95.64	95.86	96	96.6
PPV	92.31	82.5	82.5	82.67	85.0
NPV	85.08	85.28	84.6	85.01	85.0
Accuracy	85.99	84.77	84.23	84.62	84.9
**All Node**					
Sensitivity	47.37	57.43	48.33	50.91	51.0
Specificity	99.04	98.93	99.09	99.07	99.0
PPV	93.75	95.08	95.08	94.92	94.7
NPV	86.15	86.6	84.06	85.52	85.6
Accuracy	87.04	87.96	85.56	86.81	86.8
**All HPV-positive OPC**					
Sensitivity	44.44	61.54	42.11	45.45	48.4
Specificity	99.35	99.28	99.36	99.36	99.3
PPV	92.31	94.12	94.12	93.75	93.6
NPV	91.12	93.24	87.64	89.6	90.4
Accuracy	91.21	93.33	88.21	89.95	90.7
**All HPV-negative OPC**					
Sensitivity	61.54	64.29	56.25	60	60.5
Specificity	96	96	96	96	96.0
PPV	88.89	90	90	90	89.7
NPV	82.76	82.76	77.42	80	80.7
Accuracy	84.21	84.62	80.49	82.5	83.0
**All Hypopharynx/Larynx**					
Sensitivity	32.14	44.12	42.86	44.12	40.8
Specificity	100 (9)	93.75	93.88	93.75	93.8
PPV	100 (9)	83.33	83.33	83.33	57.9
NPV	70.31	70.31	69.7	70.31	70.2
Accuracy	73.97	73.17	72.62	73.17	73.2

Key: OPC = oropharyngeal cancer.

**Table 6:** Diagnostic performance of interpretative criteria for prediction of complete response and progressive disease for indeterminate scores applied to all primary tumours, all nodal disease, HPV-positive OPC, HPV-negative OPC and hypopharynx/larynx sub-groups. () = number of indeterminate cases.

	NI-RADS (n = 91)	Porceddu (n = 25)	Hopkins (n = 13)	Deauville (n = 20)	Mean
All Primary Tumour					
Sensitivity	41	11.67	9.23	9.84	17.9
Specificity	85	94.46	97.89	95.71	93.3
PPV	41.76	28	46.15	30	36.5
NPV	85.08	85.28	84.6	85.01	85.0
Accuracy	76.38	81.56	83.3	82.17	80.9
**All Node**					
	**NI-RADS (n = 55)**	**Porceddu (n = 70)**	**Hopkins (n = 3)**	**Deauville (n = 18)**	**Mean**
Sensitivity	39.02	32.81	4.62	15.62	23.0
Specificity	93.11	85.02	100	97.55	91.9
PPV	58.18	30	100	55.56	47.9
NPV	86.15	86.6	84.06	85.52	85.6
Accuracy	82.45	76.47	84.18	84.14	81.8
**All HPV-positive OPC**					
	**NI-RADS (n = 32)**	**Porceddu (n = 9)**	**Hopkins (n = 4)**	**Deauville (n = 8)**	**Mean**
Sensitivity	42.31	56.52	4.35	21.74	31.2
Specificity	96.86	88.46	100	99.36	94.9
PPV	68.75	41.94	100	83.33	64.7
NPV	91.12	93.24	87.64	89.6	90.4
Accuracy	89.19	84.36	87.71	89.39	87.7
**All HPV-negative OPC**					
	**NI-RADS (n = 9)**	**Porceddu (n = 2)**	**Hopkins (n = 1)**	**Deauville (n = 1)**	**Mean**
Sensitivity	50	28.57	12.5	14.29	26.3
Specificity	85.71	100	100	100	85.7
PPV	55.56	100	100	100	55.6
NPV	82.76	82.76	77.42	80	80.7
Accuracy	76.32	83.87	78.12	80.65	79.7
**All Hypopharynx/Larynx**					
	**NI-RADS (n = 23)**	**Porceddu (n = 2)**	**Hopkins (n = 3)**	**Deauville (n = 2)**	**Mean**
Sensitivity	34.48	5	0	5	14.8
Specificity	77.59	97.83	93.88	97.83	91.8
PPV	43.48	50	0	50	47.8
NPV	70.31	70.31	69.7	70.31	70.2
Accuracy	63.22	69.7	66.67	69.7	67.3

Key: OPC = oropharyngeal cancer.

**Figure 4:**
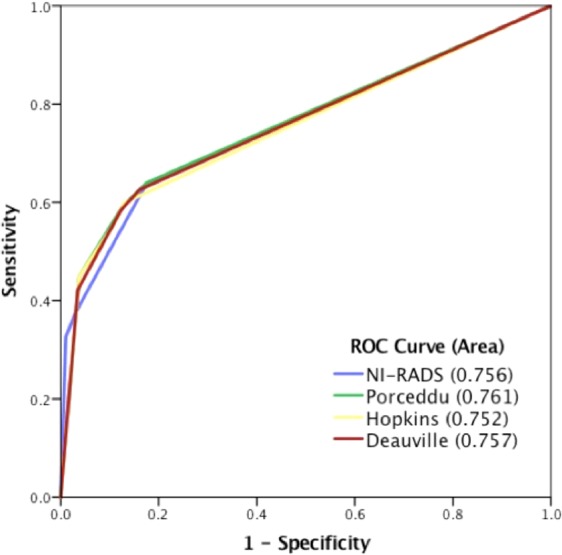
Receiver operating characteristic (ROC) curves for the four interpretative criteria.

The ROC analysis (Fig. 4) established that each of the IC were similar in their ability to predict disease outcome with areas under the curve (AUC) of 0.76 (NI-RADS), 0.76 (Porceddu), 0.75 (Hopkins) and 0.76 (Deauville) respectively.

## Discussion

The use of qualitative assessment of FDG PET-CT post treatment in HNSCC was highly predictive of PFS and OS using four previously validated criteria - NI-RADS, Porceddu, Hopkins and Deauville in our large patient cohort. All 4 adjusted IC demonstrated good discriminatory ability in predicting disease outcome with high specificity, PPV and NPV which could help clinical decision making, stratifying patients into different management streams including continued observation, biopsy or salvage surgery.

Compared to the existing literature, the PPV values of our study (83–95%) are slightly higher than other reported rates of 51–78%^14,15,23^. Diagnostic accuracy of response assessment PET-CT is affected by the time interval between treatment and follow-up imaging, the later median time-point of imaging post radiotherapy (17 weeks) compared to other studies may account for the slightly higher PPV values in this study. Conversely the NPV is lower (84–86%) compared to multiple other studies (86–97%) with smaller cohort sizes (largest 214 patients)^12,13,23–25^. PET-CT was categorised as false-negative if recurrent cancer was diagnosed at any stage during follow-up, the longest time to progression recorded was over 50 months from the end of treatment, whereas other studies limited this period to 6 months after the response assessment PET (14^23^, and had a higher NPV. A comparable study assessing Deauville criteria for nodal response assessment post CRT in 105 HNSCC patients using the same methodology for false-negatives (any time during follow-up) had a similar NPV (86.4%) (13). By restricting false negatives to those with recurrence developing within 6 months, the NPV of NI-RADS as an example, increases from 85% to 94% in our cohort.

There was greater variation in the number of cases classified as indeterminate between different IC, with far more scores in this category when applying the NI-RADS IC. This likely reflects the subjective nature of the NI-RADS indeterminate group which includes all cases which have focal mild to moderate mucosal FDG uptake without giving a reference area of uptake such as the IJV (Hopkins) or mediastinum (Deauville) thereby making it more difficult to split these cases up compared to the other IC^17,26^. The overall mean recurrence rate of 53% (range 42–69%) in NI-RADS category 2 (low suspicion for recurrence) patients in this study is also much higher than previously reported research study figures of 17.2%, highlighting that more work in large cohort studies is required to validate this^26^. One advantage identified for the Hopkins IC is the low number of indeterminate cases however the NPV was lower, particularly for HPV-positive (87.6%) and HPV-negative (77.4%) groups. Porceddu and Deauville provided the best trade off minimising indeterminate scores whilst maintaining a high NPV. Individual centres should apply one IC consistently across all patients to facilitate more standardised reporting and allow for future comparisons between institutions.

Interestingly, the NPV for HPV-positive OPC patients was higher than for the HPV-negative sub-group. Fakhry *et al*. previously reported that HPV-positive status was a good prognostic indicator with better CRT sensitivity and patient outcome^27^. This is relevant in indeterminate cases, where use of these IC may provide more information on guiding optimal management between neck dissection or surveillance. Previous research has demonstrated no association between HPV status and other semi-quantitative imaging markers in relation to predicting recurrence^28^. The higher NPV in HPV-positive patients may be potentially useful for clinicians when considering additional treatments such as neck dissection.

The prognostic value of PET is more uncertain when FDG uptake is equivocal/indeterminate across all four IC, with a low PPV, although this observation is limited by a relatively low number of cases fitting this sub-group with a median number of 22 for all tumour and node cases although this group was as low as one in the HPV status and hypopharynx/larynx subgroup analysis. The ability to more accurately distinguish between benign post-treatment inflammation or residual disease remains of paramount clinical importance as each scenario would require significantly different patient management. In longitudinal PET studies assessing lymphoma, equivocal scans have proved to represent a good rather than bad prognosis^29^. In the meantime, as advocated by the IC such as NI-RADS, indeterminate cases may be best followed up non-invasively with imaging in the form of a contrast-enhanced CT or PET^17^. One option is to perform a second interval PET-CT response assessment. Porceddu *et al*. recommend a further repeat PET-CT 4–6 weeks later (16 weeks post treatment) if the first one shows indeterminate response, with no subsequent cases of nodal failure^12^. Similarly a recent publication from our group highlighted that a second-look PET-CT 13 weeks median duration from the first response assessment PET-CT (median 30 weeks post treatment) found the majority of incomplete response cases convert to a complete metabolic response^30^. Follow-up imaging at an earlier time point results in a higher number of false positive results^31^. This warrants future evaluation in a larger prospective cohort.

Inter-observer agreement of IC was not assessed in this study mainly because previous work has shown these IC to be highly reproducible^14,16^. Limitations include the retrospective study design, heterogenous patient cohort with different sites of HNSCC and the slight difference in treatment with the majority having CRT but a small group having radiotherapy only.

Emerging studies exploring the utility of radiomic features extracted from head and neck cancers highlight the potential for more accurate prediction of disease progression using novel imaging signatures which could be augmented by artificial intelligence techniques^32–34^. Although there is no current clinical implementation of a radiomic-based decision-support system in this clinical scenario, in the future this may emerge and could result in better patient stratification and personalization of treatment^34^. Some challenges remain ahead of this including a need for greater data transparency, multi-centre collaborations for cross-validation and to confirm reproducibility of radiomic analysis methods^34^.

## Conclusion

Assessment with FDG PET-CT post-treatment in HNSCC is accurate for prediction of complete response or disease progression. All four analysed IC have similar diagnostic performance characteristics however Porceddu and Deauville provide the best trade off minimising indeterminate scores whilst maintaining a high NPV.

## Supplementary information

Supplementary Dataset 1.

## References

[1] Jemal A (2011). Global Cancer Statistics: 2011. CA Cancer J. Clin..

[2] Cleary M. P., Grossmann M. E., Ray A. (2010). Effect of Obesity on Breast Cancer Development. Veterinary Pathology.

[3] Denaro N, Merlano MC, Russi EG (2016). Follow-up in head and neck cancer: Do more does it mean do better? A systematic review and our proposal based on our experience. Clinical and Experimental Otorhinolaryngology.

[4] Lowe Val J., Duan Fenghai, Subramaniam Rathan M., Sicks JoRean D., Romanoff Justin, Bartel Twyla, Yu Jian Q. (Michael), Nussenbaum Brian, Richmon Jeremy, Arnold Charles D., Cognetti David, Stack Brendan C. (2019). Multicenter Trial of [18F]fluorodeoxyglucose Positron Emission Tomography/Computed Tomography Staging of Head and Neck Cancer and Negative Predictive Value and Surgical Impact in the N0 Neck: Results From ACRIN 6685. Journal of Clinical Oncology.

[5] Mehanna H (2016). PET-CT Surveillance versus Neck Dissection in Advanced Head and Neck Cancer. N. Engl. J. Med..

[6] Kale H, Rath TJ (2017). Chapter 3 The Role of PET/CT in Squamous Cell Carcinoma of the Head and Neck. Semin. Ultrasound, CT MRI.

[7] Vanderhoek M, Perlman SB, Jeraj R (2012). Impact of the Definition of Peak Standardized Uptake Value on Quantification of Treatment Response. J. Nucl. Med..

[8] Larson SM (2008). Clinical Utility of 18F-FDG PET/CT in Assessing the Neck After Concurrent Chemoradiotherapy for Locoregional Advanced Head and Neck Cancer. J. Nucl. Med..

[9] Ware RE (2004). Usefulness of fluorine-18 fluorodeoxyglucose positron emission tomography in patients with a residual structural abnormality after difinitive treatments for squamous cell carcinoma of the head and neck. Head Neck.

[10] Kendi AT (2015). 18F-FDG-PET/CT parameters as imaging biomarkers in oral cavity squamous cell carcinoma, is visual analysis of PET and contrast enhanced CT better than the numbers?. Eur. J. Radiol..

[11] Murphy JD (2011). Postradiation metabolic tumor volume predicts outcome in head-and-neck cancer. Int. J. Radiat. Oncol. Biol. Phys..

[12] Porceddu SV (2011). Results of a prospective study of positron emission tomography-directed management of residual nodal abnormalities in node-positive head and neck cancer after definitive radiotherapy with or without systemic therapy. Head and Neck.

[13] Sjövall J (2016). Qualitative interpretation of PET scans using a Likert scale to assess neck node response to radiotherapy in head and neck cancer. Eur. J. Nucl. Med. Mol. Imaging.

[14] Marcus C (2014). Head and Neck PET/CT: Therapy Response Interpretation Criteria (Hopkins Criteria)–Interreader Reliability, Accuracy, and Survival Outcomes. J. Nucl. Med..

[15] Roshan S (2019). Utility of Likert scale (Deauville criteria) in assessment of Chemoradiotherapy response of primary oropharyngeal squamous cell Cancer site. Clin. Imaging.

[16] Huang YC (2019). Post-chemoradiotherapy FDG PET with qualitative interpretation criteria for outcome stratification in esophageal squamous cell carcinoma. PLoS One.

[17] Aiken AH (2018). ACR Neck Imaging Reporting and Data Systems (NI-RADS): A White Paper of the ACR NI-RADS Committee. J. Am. Coll. Radiol..

[18] Prestwich RJD, Sen M, Scarsbrook A (2014). Qualitative 18F-FDG PET/CT Response Evaluation After Chemotherapy or Radiotherapy for Head and Neck Squamous Cell Carcinoma: Is There an Equivocal Group?. J. Nucl. Med..

[19] D Prestwich RJ (2010). A single centre experience with sequential and concomitant chemoradiotherapy in locally advanced stage IV tonsillar cancer. Radiat. Oncol..

[20] Arunsingh M (2019). Accuracy of Response Assessment Positron Emission Tomography-Computed Tomography Following Definitive Radiotherapy Without Chemotherapy for Head and Neck Squamous Cell Carcinoma. Clin. Oncol..

[21] Prestwich RJ, Öksüz DO, Dyker K, Coyle C, Şen M (2011). Feasibility and efficacy of induction docetaxel, cisplatin, and 5-fluorouracil chemotherapy combined with cisplatin concurrent chemoradiotherapy for nonmetastatic stage IV head-and-neck squamous cell carcinomas. Int. J. Radiat. Oncol. Biol. Phys..

[22] Slevin F (2015). Assessment of outcomes with delayed 18F-FDG PET-CT response assessment in head and neck squamous cell carcinoma. Br. J. Radiol..

[23] Balink H (2009). 18F-FDG PET as a Routine Posttreatment Surveillance Tool in Oral and Oropharyngeal Squamous Cell Carcinoma: A ProspectiveStudy. J. Nucl. Med..

[24] Gupta T (2011). Diagnostic performance of post-treatment FDG PET or FDG PET/CT imaging in head and neck cancer: A systematic review and meta-analysis. European Journal of Nuclear Medicine and Molecular Imaging.

[25] Leung AS, Rath TJ, Hughes MA, Kim S, Branstetter BF (2016). Optimal timing of first posttreatment FDG PET/CT in head and neck squamous cell carcinoma. Head and Neck.

[26] Krieger DA (2017). Initial performance of NI-RADS to predict residual or recurrent head and neck squamous cell carcinoma. Am. J. Neuroradiol..

[27] Fakhry C (2008). Improved survival of patients with human papillomavirus-positive head and neck squamous cell carcinoma in a prospective clinical trial. J. Natl. Cancer Inst..

[28] Huang YT, Kumar ASR, Bhuta S (2015). 18F-FDG PET/CT as a semiquantitative imaging marker in HPV-p16-positive oropharyngeal squamous cell cancers. Nucl. Med. Commun..

[29] Hutchings M (2006). FDG-PET after two cycles of chemotherapy predicts treatment failure and progression-free survival in Hodgkin lymphoma. Blood.

[30] Prestwich Robin J. D., Arunsingh Moses, Zhong Jim, Dyker Karen E., Vaidyanathan Sriram, Scarsbrook Andrew F. (2019). Second-look PET-CT following an initial incomplete PET-CT response to (chemo)radiotherapy for head and neck squamous cell carcinoma. European Radiology.

[31] Rulach R (2019). 12 week PET-CT has low positive predictive value for nodal residual disease in human papillomavirus-positive oropharyngeal cancers. Oral Oncol..

[32] Wu J (2019). Integrating Tumor and Nodal Imaging Characteristics at Baseline and Mid-Treatment Computed Tomography Scans to Predict Distant Metastasis in Oropharyngeal Cancer Treated With Concurrent Chemoradiotherapy. Int. J. Radiat. Oncol. Biol. Phys..

[33] Wu, J. *et al*. Tumor Subregion Evolution-based Imaging Features to Assess Early Response and Predict Prognosis in Oropharyngeal Cancer. *J. Nucl. Med*, 10.2967/jnumed.119.230037 (2019). 10.2967/jnumed.119.230037 PMC7067523 31420498

[34] Vallières M (2017). Radiomics strategies for risk assessment of tumour failure in head-and-neck cancer. Sci. Rep..

